# Mechanism of Taohong Siwu Decoction in Alleviating PD-Related Cellular Damage based on the MAPK Signaling Pathway

**DOI:** 10.4014/jmb.2512.12030

**Published:** 2026-03-09

**Authors:** Pei Peng, Yongjun Chen, Bohong Hu, Chao Zheng, Xingcheng Dong, Ji Liang

**Affiliations:** 1 Department of Medicine Oncology, Changde Hospital, Xiangya School of Medicine, Central South University (The First People’s Hospital of Changde City), Changde 415000, Hunan, P.R. China; 2 Department of Neurology, Changde Hospital, Xiangya School of Medicine, Central South University (The First People’s Hospital of Changde City), Changde 415000, Hunan, P.R. China; 3 Department of Imaging, Changde Hospital, Xiangya School of Medicine, Central South University (The First People’s Hospital of Changde City), Changde 415000, Hunan, P.R. China; 4 Department of Orthopedics, Dushu Lake Hospital Affiliated to Soochow University (Suzhou Dushu Lake Hospital), Medical Center of Soochow University, Suzhou 215125, Jiangsu, P.R. China

**Keywords:** Taohong Siwu decoction, Parkinson’s disease, 6-OHDA, SH-SY5Y cells, MAPK signaling pathway, Neuronal apoptosis

## Abstract

This study aimed to investigate the effects of Taohong Siwu Decoction (THSWD) on apoptosis, viability, and oxidative stress status in a 6-hydroxydopamine (6-OHDA)-induced SH-SY5Y cell injury model, and to analyze the potential involvement of stress-related MAPK (p38/JNK) signaling, specifically p38 and JNK, in its cytoprotective effects. A Parkinson’s disease (PD)-related neurotoxic cell injury model was established by exposing SH-SY5Y cells with 6-OHDA. After administering different doses of THSWD, the mRNA expression levels of apoptosis-related genes were quantified by quantitative real-time PCR, apoptotic cell proportions were assessed by flow cytometry, and cell viability was evaluated using the CCK-8 assay. In parallel, intracellular oxidative stress indicators, including ROS, MDA, and antioxidant defense indicators, including SOD, CAT, and GSH, were measured. A drug-component-target-disease network was constructed using network pharmacology methods, followed by protein–protein interaction analysis and Gene Ontology and KEGG pathway enrichment analyses to identify potential signaling pathways. Western blot analysis was performed to assess the phosphorylation levels of p38 and JNK proteins, and mechanistic validation was further conducted using the p38 inhibitor BIRB796. Under 6-OHDA-induced SH-SY5Y cell injury conditions, THSWD treatment significantly downregulated the mRNA expression of *BAX* and *CASPASE-3*, reduced apoptotic cell proportions, and decreased inflammatory cytokine levels, while markedly improving cell viability. Meanwhile, THSWD alleviated oxidative stress imbalance induced by 6-OHDA, as evidenced by reduced ROS and MDA levels, along with restored SOD and CAT activities, and GSH content. Network pharmacology analysis revealed that THSWD-associated candidate targets were significantly enriched in the MAPK signaling pathway. Western blot results demonstrated that THSWD significantly suppressed 6-OHDA-induced phosphorylation of p38 and JNK proteins. Furthermore, BIRB796 exhibited effects consistent with THSWD in attenuating oxidative stress, inhibiting stress-activated MAPK signaling, reducing apoptosis, and enhancing cell viability, with enhanced protective effects observed upon combined treatment. THSWD attenuates 6-OHDA-induced SH-SY5Y cell injury under PD–related neurotoxic conditions. Its cytoprotective effects may be mediated, at least in part, through regulation of the p38/JNK-related MAPK signaling pathway.

## Introduction

Parkinson’s disease (PD) is a progressive neurodegenerative disorder affecting more than six million individuals worldwide and represents the second most prevalent neurodegenerative disease after Alzheimer’s disease [1]. It predominantly occurs in older adults and is characterized by both motor and non-motor symptoms that substantially impair quality of life [2-4]. Multiple pathological mechanisms, including oxidative stress, mitochondrial dysfunction, neuroinflammation, and apoptosis of dopaminergic neurons, are implicated in the development and progression of PD [5]. Although current pharmacological and device-based therapies can alleviate clinical symptoms, no disease-modifying treatment is available to halt or reverse disease progression [6-10]. Thus, the precise molecular mechanisms underlying PD remain incompletely understood.

Accumulating evidence indicates that several intracellular signaling pathways, including the JAK2/STAT3 and mitogen-activated protein kinase (MAPK) pathways, are involved in the regulation of inflammatory responses and apoptosis in PD [5]. Among these, the MAPK pathway, a family of serine/threonine protein kinases, plays a crucial role in regulating cell differentiation, proliferation, apoptosis, and inflammatory responses [5]. Its major subfamilies—c-Jun N-terminal kinase (JNK), extracellular signal-regulated kinase (ERK), and p38 kinase—are particularly important in the initiation and progression of PD and are known to be activated under oxidative stress conditions [5]. Aberrant activation of these kinases is closely associated with neuronal injury. Specifically, sustained activation of JNK and p38 has been directly linked to dopaminergic neuron damage, oxidative stress, and mitochondrial dysfunction, serving as key contributors to PD-like pathological phenotypes [11-14]. In contrast, excessive activation of the ERK pathway has been associated with motor dysfunction in the striatum in PD models [5]. Therefore, targeting the p38/JNK MAPK signaling pathway to suppress its excessive activation may represent a promising strategy for the development of neuroprotective interventions in PD.

Traditional Chinese Medicine (TCM) has attracted increasing attention as a multicomponent, multitarget therapeutic approach, which may be particularly suitable for complex disorders such as PD [15, 16]. Taohong Siwu Decoction (THSWD), composed of Radix Angelicae Sinensis, Radix Rehmanniae Praeparata, Rhizoma Chuanxiong, Radix Paeoniae Alba, Semen Persicae, and Flos Carthami, is a classical TCM prescription traditionally used to promote blood circulation, remove blood stasis, and harmonize qi and blood [17-20]. Emerging evidence suggests that THSWD and its active constituents can modulate oxidative stress and inflammatory signaling in models of neural injury [21]. However, whether THSWD can effectively alleviate PD-related neuronal damage, particularly through modulation of stress-related MAPK signaling pathways, remains insufficiently explored and lacks systematic experimental validation. Clarifying whether THSWD exerts neuroprotective effects through this pathway is therefore of significant mechanistic relevance.

Notably, 6-hydroxydopamine (6-OHDA) is widely used as a neurotoxic agent in experimental PD models [22]. It selectively accumulates in dopaminergic neurons and induces excessive production of reactive oxygen species, leading to mitochondrial dysfunction, neuroinflammation, and subsequent dopaminergic neuronal injury [5, 22]. In the present study, a 6-OHDA-induced SH-SY5Y cell injury model was employed to investigate the effects of THSWD on PD-related apoptosis, cell viability, and oxidative stress status. In addition, we aimed to determine whether the cytoprotective effects of THSWD are mediated through regulation of the p38/JNK MAPK signaling pathway. By integrating network pharmacology predictions with experimental validation, this study seeks to provide new mechanistic evidence elucidating how THSWD alleviates PD-related neurotoxicity.

## Materials and Methods

### Drug-Component-Target-Disease Network Construction

Using the keywords “Radix Rehmanniae Praeparata,” “Radix Angelicae Sinensis,” “Radix Paeoniae Alba,” “Rhizoma Chuanxiong,” “Semen Persicae,” and “Flos Carthami,” active compounds and potential targets of the six herbal components of THSWD were retrieved from the Traditional Chinese Medicine Systems Pharmacology Database and Analysis Platform (TCMSP, http://lsp.nwu.edu.cn/tcmsp.php). The UniProt database (https://www.uniprot.org/) was used to standardize and annotate target genes. PD–related genes were obtained from GeneCards (https://www.genecards.org/) using “Parkinson’s disease” as the keyword. The overlapping genes between the herbal targets and PD-related genes were identified and imported into Cytoscape 3.8.0 to construct the drug–component–target–disease network.

### Core Gene Screening

Candidate genes were uploaded to the STRING database (https://string-db.org/) for protein-protein interaction (PPI) analysis. The resulting PPI network and node attribute files were exported, and node degree statistics were analyzed using R software. Genes with the highest degree of connectivity were defined as the core targets of the network.

### GO and KEGG Functional Enrichment Analyses

Gene Ontology (GO) and Kyoto Encyclopedia of Genes and Genomes (KEGG) enrichment analyses of the candidate genes were performed using the ClusterProfiler package in R (http://www.bioconductor.org/packages/ClusterProfiler/). The ggplot2 package was used to visualize the KEGG enrichment results. GO analysis included three categories—biological process (BP), cellular component (CC), and molecular function (MF) with a significance threshold of *P* < 0.05. KEGG pathway analysis was performed to identify significantly enriched signaling pathways (*P* < 0.05).

### THSWD

THSWD (63 g total) was composed of Radix Rehmanniae Praeparata (15 g), Radix Angelicae Sinensis (15 g), Radix Paeoniae Alba (10 g), Rhizoma Chuanxiong (8 g), Semen Persicae (9 g), and Flos Carthami (6 g). The herbs were decocted twice with 100 ml of distilled water, first for 30 min and then for 20 min. The combined filtrates were concentrated to a ratio equivalent to 1 g of crude drug per 1 ml of decoction, filtered through a 0.22-μm membrane, and stored at −20°C. The stock decoction was diluted with culture medium to yield high (20% v/v), medium (10% v/v), and low (5% v/v) concentrations, designated as THSWD-H, THSWD-M, and THSWD-L, respectively. These were added to the cells for 24 h to assess mRNA expression and cellular functions [23].

### Cell Culture and *In Vitro* Model Construction

Human neuroblastoma SH-SY5Y cells were obtained from the Shanghai Cell Bank of the Chinese Academy of Sciences. Cells were cultured in DMEM (Gibco, USA) supplemented with 10% fetal bovine serum (FBS; Gibco) at 37°C in a humidified atmosphere containing 5% CO_2_. The culture medium was replaced every 2–3 days, and cells were subcultured upon reaching 80–90% confluence. Following 24 h of culture in 1% FBS, cells were exposed to 100 μM 6-OHDA (Sigma, USA; Cat. No. 162957) to establish a PD-like injury model. After 24 h of treatment, cell viability and apoptosis were assessed, and mRNA and protein expression were analyzed. For the THSWD and MAPK inhibitor experiments, cells were first treated with 100 μM 6-OHDA for 2 h, followed by exposure to various concentrations of THSWD or 50 nM BIRB796 (Selleck; Cat. No. S1574). After 24 h of incubation, cells were collected for subsequent assays. 6-OHDA was freshly prepared and used immediately, with operations conducted in the dark. It was dissolved in serum-free medium or physiological saline containing 0.02% ascorbic acid and filtered before use [24, 25].

### CCK-8 Assay

Cell viability was evaluated using a CCK-8 kit (No. CA1210, Solarbio, China). SH-SY5Y cells in the logarithmic growth phase were seeded into 96-well plates at a density of 1 × 10^4^ cells/well and incubated for 24 h. Subsequently, 100 μM 6-OHDA was applied for 2 h, followed by treatment with three different concentrations of THSWD according to the experimental groups. Then, 10 μl of CCK-8 reagent was added to each well, and cells were incubated at 37°C for 3 h. Absorbance at 450 nm was measured using a microplate reader, and the optical density value was proportional to the number of viable cells [26].

### Flow Cytometry

After 24 h of treatment, cells were harvested using 0.25% trypsin (No. YB15050057, Yubo, China), centrifuged, and washed three times with cold phosphate-buffer saline (PBS). According to the manufacturer’s instructions for the Annexin V-FITC/PI Apoptosis Detection Kit (No. K201-100, Biovision, USA), a staining system (Annexin V-FITC/PI/binding buffer) was prepared using 1 × Annexin V binding buffer (containing Ca^2+^). Approximately 1 × 10^6^ cells were resuspended in 100 μl of staining solution, gently mixed, and incubated at room temperature for 15 min in the dark. Subsequently, 1 ml of HEPES buffer (No. PB180325, Procell, China) was added. Apoptosis was analyzed using a flow cytometer, detecting FITC and PI fluorescence at 488 nm excitation with emission filters of 525 nm (FITC) and 620 nm (PI) [27].

### RT-qPCR

Total RNA was extracted from cells using the EZ-press RNA Purification Kit (No. B0004D, EZBioscience). RNA concentration and purity were determined spectrophotometrically. cDNA synthesis was performed using the HiScript III RT SuperMix for qPCR (+gDNA wiper) (No. R323-01, Vazyme), and amplification was carried out using the ChamQ Universal SYBR qPCR Master Mix (No. Q711-02, Vazyme). The expression levels of *IL-6*, *IL-1β*, *BAX*, *BCL-2*, and *CASPASE-3* were quantified, with *GAPDH* serving as the internal reference gene. Primers were synthesized by Sangon Biotech (China) (Table 1). Relative expression levels were calculated using the 2^-ΔΔCt^ method, where ΔCt = Ct_(target gene)_ − Ct_(*GAPDH*)_.

### Western Blot Analysis

Total protein was extracted using RIPA lysis buffer containing PMSF (No. P0013C, Beyotime, China) and incubated on ice for 30 min. Lysates were centrifuged at 8,000 ×*g* for 10 min at 4°C, and supernatants were collected. Protein concentration was determined with a BCA kit (No. 23227, Thermo Fisher Scientific, USA). Equal amounts of protein (50 μg) were mixed with 2× SDS loading buffer, boiled at 100°C for 5 min, and separated on 10% SDS-PAGE gels (No. G2177-50T, Servicebio, China). Proteins were transferred to PVDF membranes (No. ISEQ00010, Millipore, USA) and blocked with 5% skim milk for 1 h at room temperature. Membranes were then incubated overnight at 4°C with primary antibodies against phospho-p38 (ab178867, 1:1000, Abcam), p38 (ab170099, 1:1000, Abcam), phospho-JNK (#4668, 1:2000, CST, USA), JNK (#9252, 1:2000, CST), and GAPDH (ab9485, 1:2500, Abcam). After washing three times with TBST (10 minutes each), membranes were incubated for 1 hour at room temperature with HRP-conjugated secondary antibody (goat anti-rabbit IgG H&L (HRP), ab97051, 1:2000, Abcam). Signals were detected using an ECL detection kit (No. abs920, Absin, China). Images were captured using a Bio-Rad imaging system, and grayscale intensity was analyzed with Quantity One v4.6.2 software. Target protein expression was normalized to GAPDH [28, 29].

### ELISA

After 24 h of treatment, culture supernatants were collected, and TNF-α secretion was quantified using a TNF-α ELISA Kit (No. hz-H0109c, eBioscience). Absorbance was measured at 450 nm and 570 nm using a SpectraMax i3 spectrophotometer. The 570 nm reading was subtracted from the 450 nm value to correct for background interference. Cytokine concentrations were calculated based on a standard curve [30].

### Detection of Intracellular ROS Level

To evaluate changes in intracellular reactive oxygen species (ROS) levels in SH-SY5Y cells under different treatment conditions, the 2′,7′-dichlorodihydrofluorescein diacetate (DCFH-DA) probe was used. After treatment according to the established groups for 24 h, the culture medium was discarded, and the cells were washed twice with serum-free DMEM. A working solution of the DCFH-DA at a final concentration of 10 μM (Cat. No. S0033S, Beyotime) was added, followed by incubation at 37°C for 30 min in the dark. After incubation, cells were thoroughly washed with serum-free medium to remove unbound probe. Fluorescence intensity was measured using a multifunctional microplate reader (SpectraMax i3x, Molecular Devices, USA) at an excitation wavelength of 488 nm and an emission wavelength of 525 nm. Results were expressed as relative fluorescence intensity to reflect intracellular ROS levels.

### Determination of Malondialdehyde (MDA) Content

After 24 h of treatment, cells were washed with cold PBS and collected. Cells were lysed on ice using cell lysis buffer (Cat. No. P0013, Beyotime). After centrifugation at 12,000 g for 10 min at 4°C, the supernatant was collected. MDA content was measured using an MDA detection kit based on the thiobarbituric acid method (Cat. No. S0131S, Beyotime) according to the manufacturer’s instructions. Absorbance was measured at 532 nm. MDA contents were expressed as nmol/mg protein, with protein concentration determined using a BCA protein assay kit (Cat. No. 23227, Thermo Fisher Scientific).

### Determination of Antioxidant Defense Indicators

To evaluate changes in cellular antioxidant defense capacity, superoxide dismutase (SOD) activity, catalase (CAT) activity, and glutathione (GSH) levels were assessed. After treatment, cells were lysed as described above. SOD activity was measured using a total superoxide dismutase assay kit (WST-8 method; Cat. No. S0101S, Beyotime), CAT activity was determined using a catalase assay kit (Cat. No. S0051, Beyotime), and GSH levels were quantified using a reduced glutathione assay kit (Cat. No. S0053, Beyotime). All assays were performed strictly according to the manufacturers’ instructions. Absorbance was read at the corresponding wavelengths using a SpectraMax i3x multifunctional microplate reader. Results were normalized to total protein content.

### Statistical Methods

All data are presented as mean ± standard deviation (SD). Comparisons between two groups were conducted using the t-test, while one-way ANOVA, followed by post-hoc tests (least significant difference for homogeneous variances and Tamhane’s T2 test for heterogeneous variances), was applied for multiple-group comparisons. Each experiment was performed four times. A *P* value < 0.05 was considered statistically significant. Statistical analyses were performed using GraphPad Prism version 6.0.

## Results

### THSWD Inhibits Neuronal Apoptosis and Inflammation in the 6-OHDA-Induced Cell Injury Model

The potential of TCM in neurodegenerative disorders has gained increasing recognition in recent years. Among various TCM formulations, THSWD has demonstrated therapeutic efficacy in multiple disease contexts. To evaluate the effect of THSWD on toxin-induced neuronal-like cell injury, SH-SY5Y cells were treated with 6-OHDA to establish a 6-OHDA-induced cell injury model. Injured cells were subsequently treated with graded concentrations of THSWD: 5% (THSWD-L), 10% (THSWD-M), and 20% (THSWD-H) of the original decoction volume.

RT-qPCR analysis showed that compared with the control group, the mRNA expression of pro-apoptotic genes (*BAX* and *CASPASE-3*) was markedly elevated, whereas *BCL-2* expression was significantly decreased in the 6-OHDA group. Conversely, THSWD treatment significantly downregulated *BAX* and *CASPASE-3* mRNA levels while upregulating *BCL-2* in a dose-dependent manner, indicating suppression of apoptosis-related signaling (Fig. 1A). Flow cytometry revealed a substantial reduction in the percentage of apoptotic cells following THSWD treatment, with the medium-dose group exhibiting the most pronounced anti-apoptotic effect (Fig. 1B and 1C). Consistently, the CCK-8 assay demonstrated that cell viability, which was severely impaired by 6-OHDA, was significantly restored upon THSWD administration, most prominently in the medium-dose group (Fig. 1D). ELISA analysis showed a pronounced increase in TNF-α secretion in the 6-OHDA group, which was markedly reduced after THSWD treatment (Fig. 1E). Similarly, RT-qPCR revealed that the mRNA expression of *IL-6* and *IL-1β* was significantly elevated in the 6-OHDA group but declined markedly after THSWD exposure (Fig. 1F). Collectively, these results indicate that THSWD inhibits neuronal apoptosis, preserves cell viability, and alleviates inflammatory responses. Notably, no significant difference was observed between the THSWD-alone treatment group and the Control group, suggesting that THSWD does not exert detectable cytotoxic effects under the experimental conditions.

Taken together, THSWD demonstrates consistent cytoprotective effects against 6-OHDA-induced SH-SY5Y cell injury, primarily manifested by reduced apoptosis-related gene expression, decreased apoptotic cell proportion, improved cell viability, and attenuated inflammatory responses.

### Candidate Target Genes of THSWD Involved in PD Are Enriched in the MAPK Signaling Pathway

To elucidate the potential molecular mechanisms underlying the neuroprotective effects of THSWD, active components and putative targets were retrieved from the TCMSP database based on its six herbal constituents: Radix Rehmanniae Praeparata, Radix Angelicae Sinensis, Radix Paeoniae Alba, Rhizoma Chuanxiong, Semen Persicae, and Flos Carthami. A total of 150 drug-related targets were identified. Searching the GeneCards database using the keyword “Parkinson’s disease” yielded 132 PD-associated genes. Cross-analysis revealed 15 overlapping genes between the two datasets (Fig. 2A). These targets were subsequently used to construct the drug–component–target–disease interaction network in Cytoscape (Fig. 2B).

Fifteen candidate genes potentially targeted by THSWD were identified. To further determine the core regulatory genes among them, these candidates were imported into the STRING database for PPI analysis (Fig. 3A) and ranked according to Degree value, representing the number of connections between a given node and other nodes. *MAPK1* and *MAPK14* exhibited relatively high connectivity (Fig. 3B). It should be noted that *MAPK1* (encoding ERK2) belongs to the ERK branch, while *MAPK14* (encoding p38α) and JNK are members of the stress-related MAPK branch and exhibit distinct biological functions. Combined with the GO and KEGG enrichment analyses, the candidate targets were significantly enriched in MAPK signaling-related pathways (Fig. 3C and 3D). Given that the observed phenotypes in this study were mainly associated with stress, inflammation, and apoptosis, subsequent experimental validation focused on the activation status of stress-related MAPK proteins (p38 and JNK).

### THSWD Inhibits 6-OHDA-Induced Phosphorylation of Stress-Related MAPK (p38/JNK)

The MAPK (p38) signaling cascade regulates diverse cellular processes, including proliferation, differentiation, stress response, inflammation, and apoptosis [31]. In neurodegenerative diseases such as PD, aberrant activation of MAPK signaling—particularly phosphorylation of p38 and JNK—has been closely associated with neuronal injury and disease progression [32].

Western blot analysis showed that phosphorylation levels of JNK and p38 were significantly increased in 6-OHDA-injured SH-SY5Y cells compared with the control group. Treatment with medium-dose THSWD markedly reduced the phosphorylation of both kinases (Fig. 4A and 4B). This suggests that THSWD inhibits 6-OHDA-induced activation of stress-related MAPK signaling.

### The p38/JNK Signaling Pathway Is Involved in the Cytoprotective Effects Mediated by THSWD

To further evaluate the potential involvement of stress-activated MAPK signaling in the cytoprotective effects of THSWD, the p38 inhibitor BIRB796 was combined with medium-dose THSWD (THSWD-M) in the 6-OHDA injury model. Western blot results demonstrated that phosphorylation levels of p38 and JNK were significantly decreased in both the 6-OHDA + BIRB796 and 6-OHDA + THSWD-M groups relative to the 6-OHDA + DMSO group. The combined treatment with THSWD-M and BIRB796 produced the most pronounced suppression of MAPK phosphorylation (Fig. 5A). Consistently, RT-qPCR results showed that the mRNA expression of the pro-apoptotic genes *BAX* and *CASPASE-3* was markedly downregulated, while *BCL-2* expression was upregulated in both the THSWD-M and BIRB796 groups, with the strongest effect observed in the combined treatment group (Fig. 5B and 5C). The CCK-8 assay further confirmed enhanced cell viability following either THSWD-M or BIRB796 treatment, with a more pronounced improvement observed when both treatments were combined (Fig. 5D).

Given that oxidative stress was an important upstream event of 6-OHDA-induced neurotoxicity and stress-related MAPK activation, oxidative stress-related indicators were further assessed in SH-SY5Y cells. Compared with the 6-OHDA + DMSO group, intracellular ROS and MDA levels were reduced in the 6-OHDA + THSWD-M + DMSO group and the 6-OHDA + THSWD-M + BIRB796 group, while SOD and CAT activities were increased and GSH levels were partially restored. In the 6-OHDA + THSWD-M + BIRB796 group, ROS and MDA levels further decreased, and SOD and CAT activities and GSH levels were further restored (Fig. 5E-5I).

Taken together, THSWD reduces apoptosis, improves cell viability, and alleviates oxidative stress in the 6-OHDA–induced SH-SY5Y cell injury model. These phenotypic changes are consistent with inhibition of stress-related p38/JNK MAPK signaling, suggesting that this pathway contributes to the cytoprotective effects of THSWD, although it is unlikely to represent the sole mechanism of action.

## Discussion

This study found that THSWD alleviates 6-OHDA-induced SH-SY5Y cell injury, as evidenced by reduced oxidative stress levels, decreased cell apoptosis, and improved cell viability. Moreover, these protective effects are associated with the inhibition of activation of stress-related MAPK (p38/JNK) signaling pathways.

Firstly, the results showed that THSWD inhibited neuronal apoptosis and inflammation in a 6-OHDA-induced cell injury model, and exhibited no detectable cytotoxicity, as no significant difference was observed between the THSWD-alone group and the Control group. These findings are consistent with the multi-target properties of compound TCM as a cellular stress regulator [33, 34]. Wang *et al*. reported that THSWD exerts anti-inflammatory effects by intervening in inflammatory signaling networks through multiple targets [35]. Han *et al*. showed that THSWD suppresses oxidative stress–induced cardiomyocyte apoptosis both *in vitro* and *in vivo* by modulating the PTEN-related signaling pathway [36]. In the present study, network pharmacology analysis further indicated that candidate targets of THSWD involved in PD regulation were enriched in the MAPK signaling pathway. The MAPK cascade represents a central intracellular regulatory network governing cell growth, differentiation, stress adaptation, and apoptosis [37].

We further demonstrated that THSWD treatment inhibited 6-OHDA–induced phosphorylation of p38 and JNK proteins. This inhibitory effect was closely associated with the cytoprotective actions of THSWD. Specifically, THSWD reduced intracellular ROS and MDA levels while restoring SOD and CAT activities and GSH content, thereby attenuating oxidative damage induced by 6-OHDA. These findings are consistent with the established mechanism whereby 6-OHDA induces cellular injury through auto-oxidation–mediated ROS generation [22]. Pharmacological experiments further showed that the p38 inhibitor BIRB796 synergistically enhanced the anti-apoptotic and antioxidant effects of THSWD, accompanied by more pronounced suppression of p38/JNK phosphorylation. Collectively, these data indicate that inhibition of oxidative stress–triggered p38/JNK activation is an important mechanism underlying the protective effects of THSWD.

Accumulating evidence indicates that THSWD reduces excessive ROS production in cerebral ischemia–reperfusion injury by activating mitochondrial quality control mechanisms, thereby blocking ROS-driven neuroinflammatory pathways and exerting neuroprotective effects [38]. In neural injury models, the p38/JNK pathway represents a key downstream mediator of oxidative stress–associated neuroinflammation and injury [39]. Lu *et al*. reported that Shenghui Decoction attenuates neuroinflammation and oxidative stress by inhibiting phosphorylation of the p38/JNK MAPK signaling pathway [39]. Together with previous findings that THSWD modulates oxidative stress–related signaling pathways [36], these observations suggest that regulation of the oxidative stress–MAPK axis may represent a common mechanism by which THSWD exerts protective effects. Notably, the present study also observed that the medium-dose group showed stronger effects than the high-dose group for certain indicators, suggesting a possible non-linear dose–response relationship or an optimal effective window, potentially related to osmotic pressure, pH changes, or interactions among components at higher concentrations.

The cellular findings were consistent with network pharmacology predictions. Molecular docking analysis suggested that certain THSWD components, such as myricanone and luteolin, exhibit strong binding affinity to p38 MAPK. Myricanone derived from Ligusticum chuanxiong has been reported to alleviate neuropathic pain by targeting MAPK-related signaling [40], while luteolin exerts anti-inflammatory and antioxidant effects through modulation of signaling pathways such as NF-κB [41]. These observations provide a plausible molecular interaction basis for the protective effects of THSWD observed in this study.

Several limitations should be acknowledged. First, the 6-OHDA–induced SH-SY5Y cell injury model primarily reflects acute oxidative stress and apoptosis and does not recapitulate the complex pathophysiology of PD *in vivo*. Therefore, the observed effects should be interpreted as attenuation of toxin-induced cellular stress rather than direct evidence of disease modification. Second, MAPK signaling and apoptotic pathways are broadly involved in cellular stress responses, and pharmacological inhibition of these pathways in an *in vitro* toxin model does not establish PD-specific neuroprotection. Finally, the involvement of the MAPK pathway was inferred mainly from phenotypic consistency between THSWD treatment and MAPK inhibition, and a strict pathway-dependent causal relationship remains to be established.

In summary, this study demonstrates that THSWD alleviates SH-SY5Y cell injury under PD-related neurotoxic stress conditions, with cytoprotective effects associated with regulation of p38/JNK-related MAPK signaling. Future studies using models more representative of *in vivo* pathology, such as primary dopaminergic neurons or animal models, as well as genetic approaches targeting p38 or JNK, are warranted to further clarify the role of this pathway and delineate the underlying molecular mechanisms.

## Figures and Tables

**Fig. 1:**
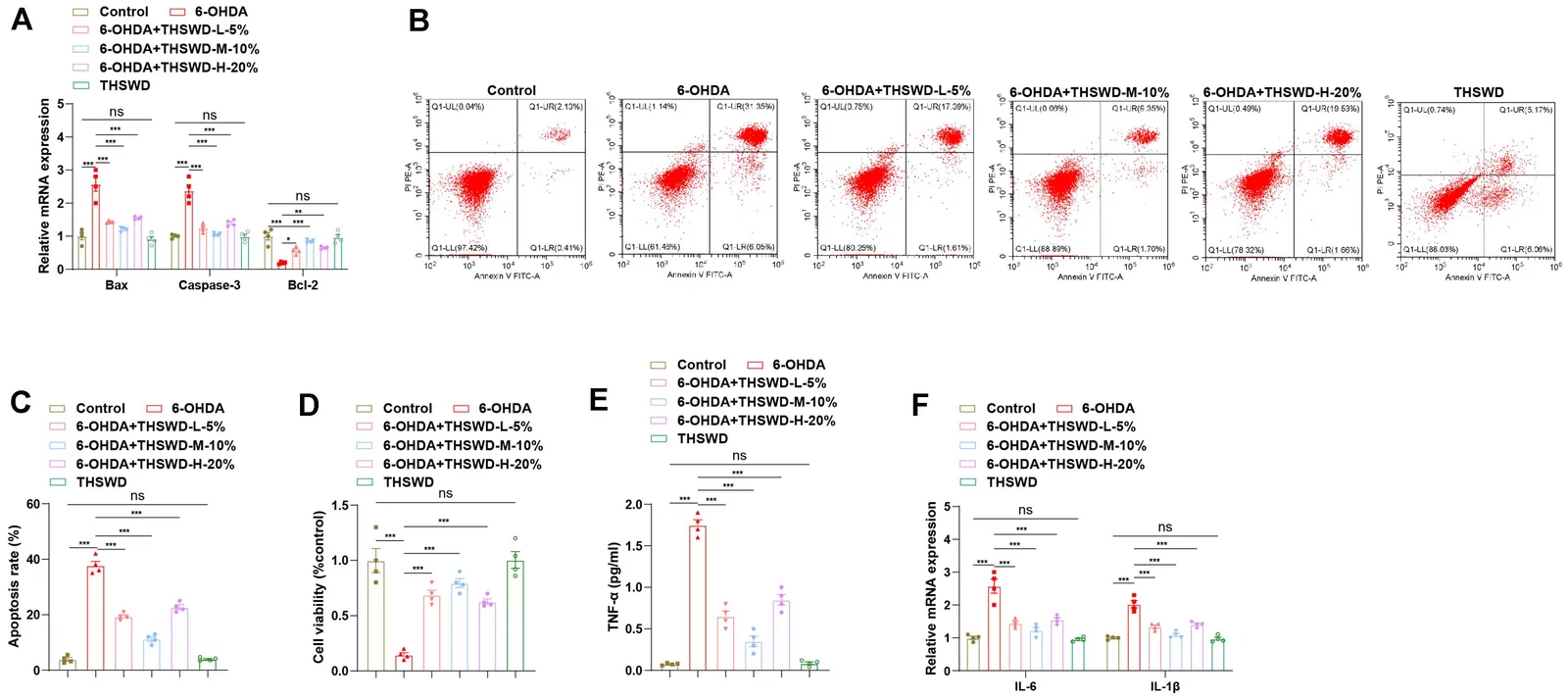
THSWD inhibits neuronal apoptosis in the PD cell model. (**A**) RT-qPCR analysis of mRNA expression levels of apoptosis-related genes in 6-OHDA-induced SH-SY5Y cell injury and after treatment with different doses of THSWD, *n* = 4; (**B-C**) Flow cytometric analysis of apoptotic cell proportion in 6-OHDA-induced SH-SY5Y cell injury and after treatment with different doses of THSWD, *n* = 4; (**D**) CCK-8 assay evaluating the effects of different doses of THSWD on cell viability in 6-OHDA-treated cells, *n* = 4; **E:** ELISA analysis of TNF-α secretion in 6-OHDA-treated cells following THSWD administration, *n* = 4; **F:** RT-qPCR analysis of mRNA expression levels of inflammation-related genes after THSWD treatment, *n* = 4; * represents *P* < 0.05 compared between two groups.

**Fig. 2:**
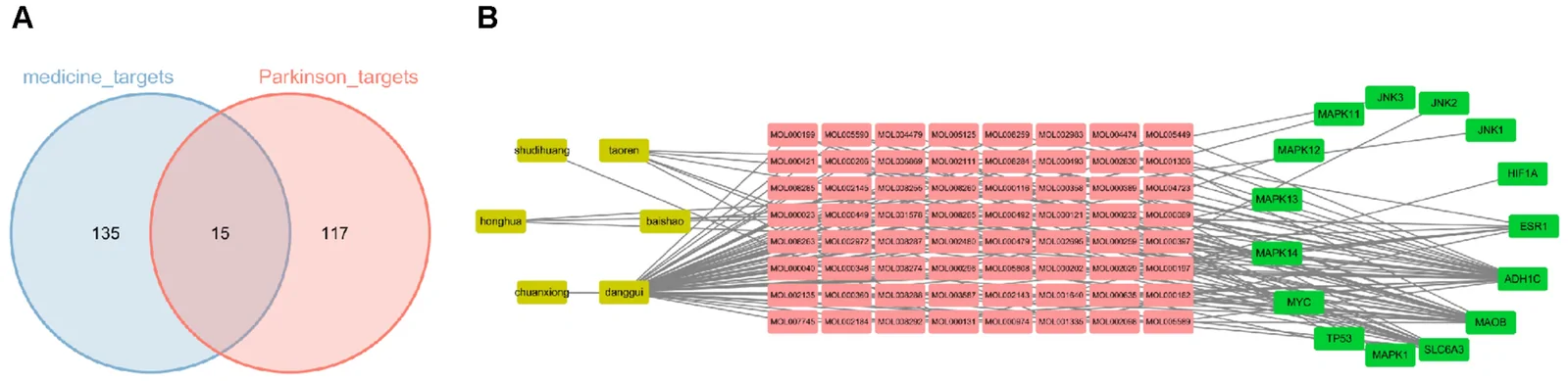
Drug-component-target-disease network construction. (**A**) Venn diagram showing the overlap between predicted THSWD targets and PD-related genes; (**B**) Drug–component–target–disease interaction network constructed using Cytoscape.

**Fig. 3:**
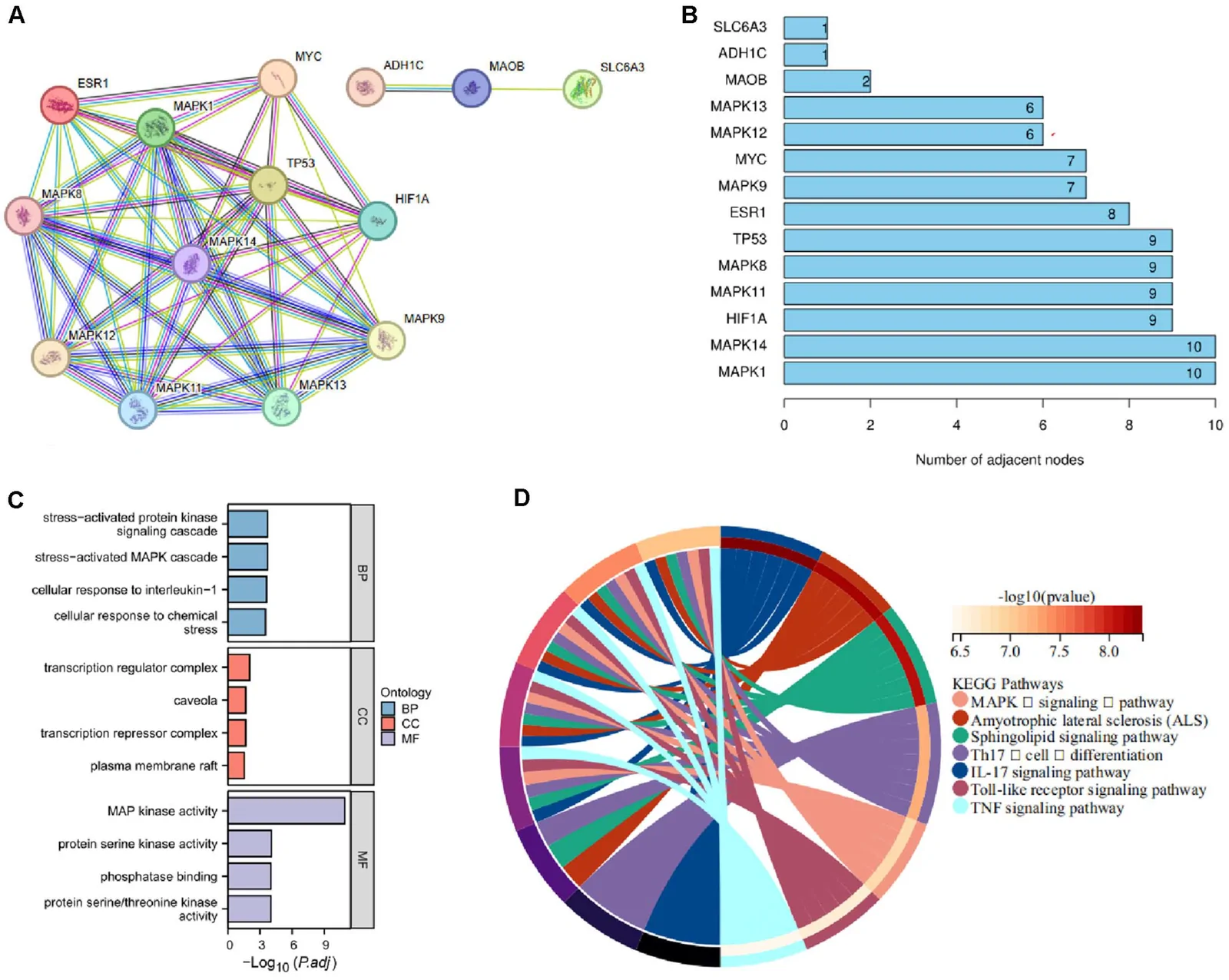
Candidate target genes of THSWD involved in PD are significantly enriched in the MAPK signaling pathway. (**A**) Protein–protein interaction network of proteins encoded by the 15 candidate targets; (**B**) Degree values of proteins encoded by the 15 candidate targets in the interaction network; (**C**) GO enrichment analysis; **D:** KEGG pathway enrichment analysis.

**Fig. 4:**
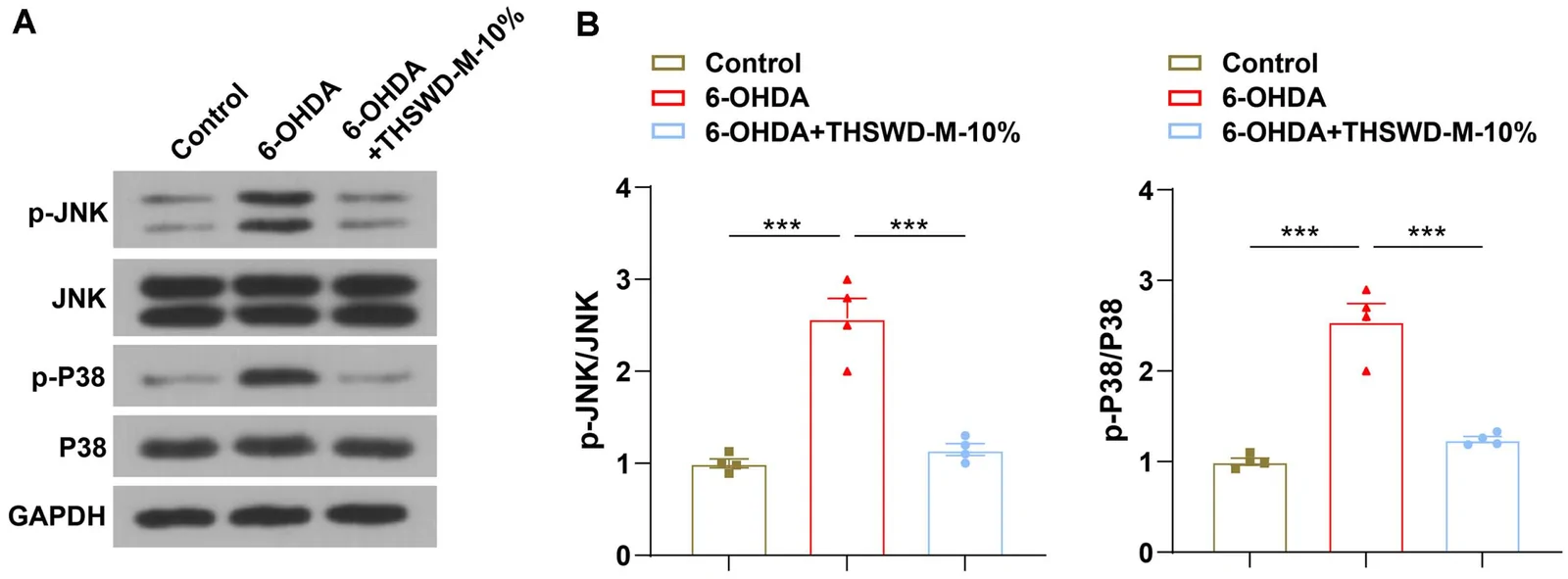
THSWD inhibits the MAPK signaling pathway. (**A-B**) Western blot assay of the effect of medium-dose THSWD (10% decoction volume) on the phosphorylation levels of p38 and JNK proteins in 6-OHDA-treated cells, *n* = 4; * represents *P* < 0.05 compared between the two groups.

**Fig. 5:**
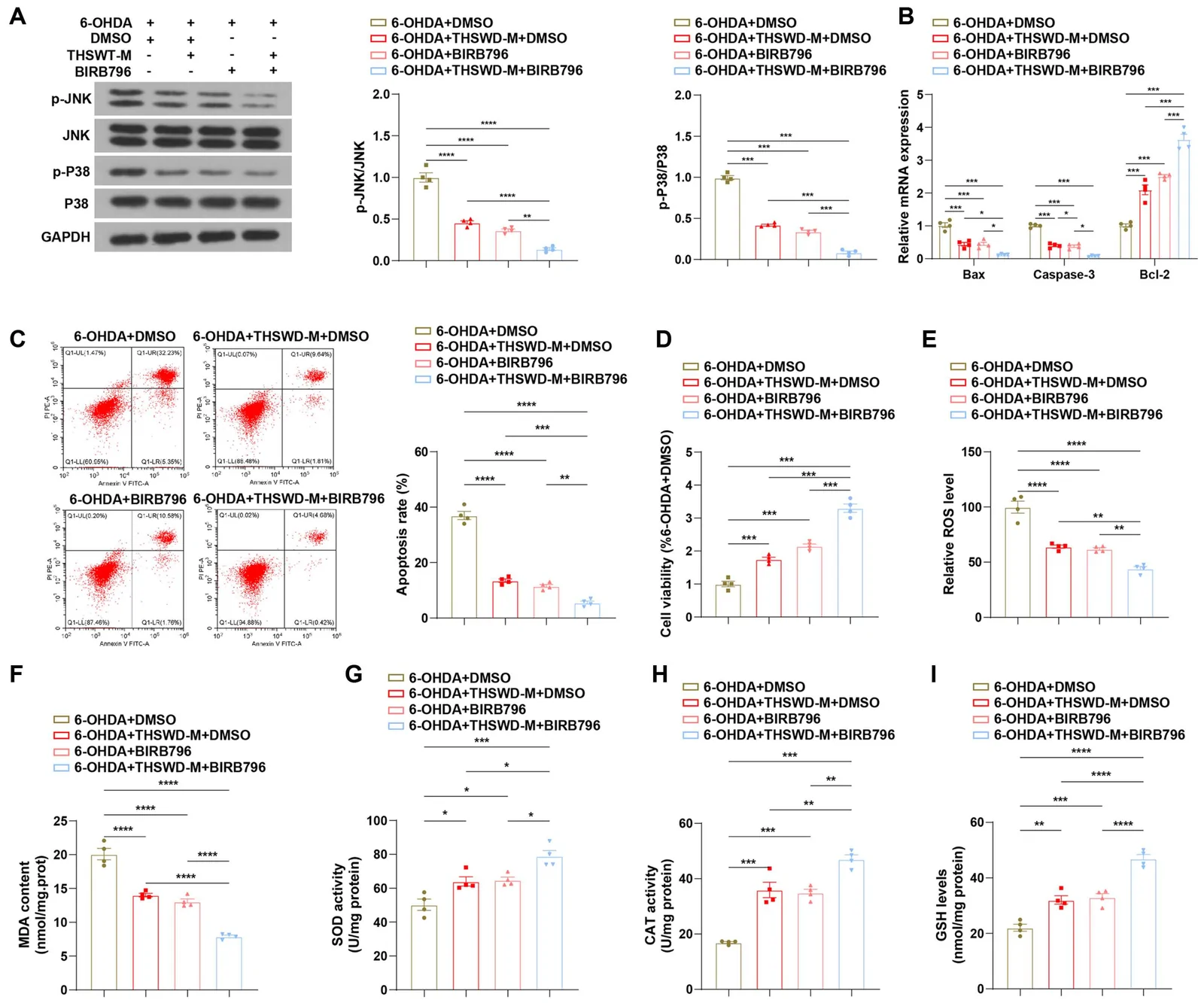
THSWD alleviates 6-OHDA-induced oxidative stress imbalance and neuronal apoptosis in SH-SY5Y cells. (**A**) Western blot analysis of p38 and JNK phosphorylation under different treatment conditions, *n* = 4; (**B**) RT-qPCR analysis of the mRNA expression levels of apoptosis-related genes, *n* = 4; (**C**) Flow cytometric analysis of apoptotic cell proportions following cell injury, *n* = 4; (**D**) CCK-8 assay assessing cell viability, *n* = 4; (**E**) DCFH-DA fluorescence probe-based detection of intracellular ROS levels in SH-SY5Y cells under different treatment conditions, *n* = 4; (**F**) Thiobarbituric acid assay for MDA content, *n* = 4; (**G-H**) Colorimetric assays for intracellular SOD and CAT activities, *n* = 4; **I:** Colorimetric assay for intracellular reduced GSH levels, *n* = 4; * represents *P* < 0.05 compared between two groups.

**Table 1:** Sequences of RT-qPCR primers.

Name	Sequence
IL-6-F	5′-CACAGACAGCCACTCACCTC-3′
IL-6-R	5′-TTTTCTGCCAGTGCCTCTTT-3′
IL-1β-F	5′-ATGGGATAACGAGGCTTATGTG-3′
IL-1β-R	5′-CAAGGCCACAGGTATTTTGTC-3′
Bax-F	5′-GGACGAACTGGACAGTAACATGG-3′
Bax-R	5′-GCAAAGTAGAAAAGGGCGACAAC-3′
Bcl-2-F	5′-ATCGCCCTGTGGATGACTGAG-3′
Bcl-2-R	5′-CAGCCAGGAGAAATCAAACAGAGG-3′
Caspase-3-F	5′-TCCACAGCACCTGGTTATTA-3′
Caspase-3-R	5′-AGGACTCAAATTCTGTTGCCA-3′
GAPDH-F	5′-GGAGCGAGATCCCTCCAAAAT-3′
GAPDH-R	5′-GGCTGTTGTCATACTTCTCATGG-3′

F, forward; R, reverse.
